# Intravenous Lipid Emulsion for treating Tramadol-Induced Seizures: Surprising but Worth Considering for Future Studies; a Letter to Editor

**DOI:** 10.22037/aaem.v10i1.1541

**Published:** 2022-02-09

**Authors:** Bruno Mégarbane, Ahmed S. Gouda

**Affiliations:** 1 Department of Medical and Toxicological Critical Care, Lariboisière Hospital; Paris, University; INSERM UMRS-1144, Paris, France.; 2 National Egyptian Center for Toxicological Researches, Faculty of Medicine, University of Cairo, Cairo, Egypt.

**Dear Editor;**

We read with interest the article reporting benefits of intravenous lipid emulsion (ILE) in preventing tramadol-induced in-hospital seizures in poisoned patients (1). We would first like to congratulate the authors for this impressive randomized investigation. However, we wish to comment on their findings.

The main presumed mechanism of action for ILE in acutely poisoned patients, named “lipid sink”, is limiting tissue distribution of lipophilic drugs potentially causing toxicity. Based on an *in vitro* model, ILE’s ability to sequester a drug was shown to parallel its octanol-water partition coefficient (2), defined as the ratio of its concentration in a water-saturated octanolic phase to its concentration in an octanol-saturated aqueous phase and usually expressed as “LogP”. Prediction of drug binding with ILE was additionally improved by combining its LogP with volume of distribution (V_D_), together accounting for ~88% of ILE-attributed variation in its serum concentration decrease (2). With a LogP of 1.34 (3), only ~1% decrease in serum tramadol level should be expected with ILE. By combining its LogP and V_D_ of 2.7L/kg (3), predicted lipid extraction efficiency would also remain extremely low (<10%), precluding any important clinical benefit. Noteworthy, this issue would have been different in case of much more lipophilic opioids such as fentanyl (LogP of 4.05 and V_D_ of 4L/kg), with a predicted ILE-related decrease in serum concentration of >40% (2, 3).

Another issue when considering ILE to reverse toxicity after oral exposure is the delay between ingestion and hospital admission, which is important since patients are usually referred after the onset of the first toxic symptoms. Management commonly starts at a time in the poisoning course when the drug distribution phase is almost complete. However, ILE’s ability to redistribute the toxicant from its target organs is much more controversial. 

Although tramadol causes a high risk of seizure, seizure event rate in tramadol-overdosed patients was determined at 38% (95%CI: 27- 49) versus 37% (95%CI: 12 - 62) in tramadol abusers and 3% (95%CI: 2 - 3) in users with therapeutic dosage (4). Seizure risk is dose-dependent, more marked in patients with existing seizure disorders or previous seizure history. Various co-ingestions such as serotonergic medications may increase the risk, while benzodiazepines, frequently used in addicts, may decrease it. These observations suggest that one in three tramadol-exposed patients admitted to the emergency department is actually at risk of seizing. In Kazemifar’s trial, 56% of the included patients seized before receiving one of the two arm treatments and 37% of the controls presented in-hospital seizures (1). In this view, patients included in the study appeared at an unusually elevated risk of seizing, especially given the relatively low mean ingested tramadol dose reported with a huge standard deviation (229 ± 135 mg in the intervention and 195 ± 156 mg in the control group, whereas the pharmacological single dose is 100 mg and the daily dose 400 mg).

Tramadol typically results in early, single, brief, self-limiting generalized tonic-clonic seizures, often occurring within 4-6h post-ingestion (5). Multiple seizure risk is also low (6). Clinical toxicologists even stated the lack of need to administer anticonvulsants for seizure prophylaxis beyond that required for treating ongoing convulsions or other co-morbidities in tramadol-poisoned patients who presented an initial seizure (6). Interestingly, a decision tree based on readily available parameters on admission was published in the journal to help clinicians identifying tramadol-poisoned patients at high risk for seizure (area under the ROC curve = 0.77; 95%CI: 0.67–0.87) to possibly treat them with prophylactic anticonvulsants (7).

Current recommendations on ILE use were published by an international collaborative workgroup supported by the American and European societies of clinical toxicology (8). When managing life-threatening toxicity, guidelines recommended not using ILE as first-line therapy. As part of treatment modalities and if other therapies fail, guidelines recommended using ILE only in bupivacaine toxicity, but suggested using ILE for toxicity due to other local anesthetics, amitriptyline, and bupropion. Recommendations were neutral for all other toxins and variable for the management of non-life-threatening toxicity, relying on the expected risk/benefit balance for each involved toxicant. 

Despite possible biases and surprising results, Kazemifar’s findings (1) clearly question whether tramadol-induced seizure prophylaxis should be added to the list of ILE indications. Alternatively, in tramadol-poisoned patients at risk of seizures or respiratory depression, more specific antidotes, i.e., naloxone, diazepam, or their combination, might also be considered, as suggested in a rat model (9). Therefore, to improve tramadol-poisoned patients’ management, further randomized studies with better design and larger cohorts, comparing ILE with alternative therapeutic strategies should be considered.

## 1. Declarations:

### 1.1 Financial Disclosure

The authors declare no financial interests in relation to the manuscript.

### 1.2 Funding support

None declared.

### 1.3 Authors’ Contributions

Bruno Mégarbane wrote the first draft. Ahmed S. Gouda commented on the draft. Both authors agreed with the final version of the manuscript.

### 1.4 Acknowledgment

None.
